# ﻿*Schnabeliajiuzhaigouensis* (Lamiaceae, Ajugoideae), a new species from Sichuan, China

**DOI:** 10.3897/phytokeys.253.141697

**Published:** 2025-02-26

**Authors:** Fei Zhao, Peng-Wei Gao, Ting Zhang, Cheng Liu, Chun-Lei Xiang

**Affiliations:** 1 CAS Key Laboratory of Mountain Ecological Restoration and Bioresource Utilization & Ecological Restoration and Biodiversity Conservation Key Laboratory of Sichuan Province, Chengdu Institute of Biology, Chinese Academy of Sciences, Chengdu 610213, Sichuan, China; 2 CAS Key Laboratory for Plant Diversity and Biogeography of East Asia, Kunming Institute of Botany, Chinese Academy of Sciences, Kunming 650201, Yunnan, China; 3 College of Life Sciences, University of Chinese Academy of Sciences, Beijing 100049, China; 4 Germplasm Bank of Wild Species, Kunming Institute of Botany, Chinese Academy of Sciences, Kunming 650201, Yunnan, China; 5 State Key Laboratory of Phytochemistry and Natural Medicines, Kunming 650201, Yunnan, China

**Keywords:** Ajugeae, morphology, new species, *Schnabelia*, taxonomy

## Abstract

*Schnabeliajiuzhaigouensis* C. Liu, F. Zhao & C.L. Xiang, a new species from north-eastern Sichuan, China, is described, based on both morphological and molecular phylogenetic evidence. Morphologically, the new species is mostly similar to *S.terniflora*, but can be readily distinguished by its habit, ovate to lanceolate-ovate leaf blades, the presence of 5–8 lateral veins on each side of the mid-rib and distinct characteristics of the floral structure, including oblong upper lip and lateral lobes of the lower lip, with the middle lobe being cuneate. In addition, the nutlets are puberulent and lack reticulate venation. Molecular phylogenetic analyses place the new species at a basal position within the genus *Schnabelia*. The new species is categorised as Data Deficient (DD) according to the IUCN Red List Categories and Criteria. A key to all species of *Schnabelia* is also provided.

## ﻿Introduction

*Schnabelia* Hand.-Mazz. (Lamiaceae, Ajugoideae) is a small genus endemic to China. The genus was first described by Handel-Mazzetti (1921), based on specimens collected from Hunan Province in central China. The taxonomic placement and circumscription of *Schnabelia* have long been debated. Initially, in the traditional taxonomic treatment (Handel-Mazzetti 1921; Chen 1977; Chen and Gilbert 1994), the genus was placed within Verbenaceae, containing only two species (*S.oligophylla* Hand.-Mazz. and *S.tetrodonta* (Y.Z.Sun) C.Y.Wu & C.Chen), both characterised by 4-winged stems. However, based on the character of the deeply-lobed ovaries, P’ei (1932) transferred *Schnabelia* to the family Lamiaceae, associating it with *Ajuga* L. and *Teucrium* L., a classification supported by P’ei and Chen (1977). Cladistic analysis using morphological data by Cantino et al. (1999) revealed that the traditionally defined *Caryopteris* Bunge was not monophyletic and led to the transfer of three species of *Caryopteris* (i.e. *C.aureoglandulosa* (Vaniot) C.Y.Wu, *C.nepetifolia* (Benth.) Maxim. and *C.terniflora* Maxim) to *Schnabelia*. Subsequent molecular phylogenetic analyses (Huang 2002; Shi et al. 2003) further confirmed that at least the latter two species are closely related to *S.oligophylla*.

Recently, based on broad sampling and comprehensive evidence from morphological and molecular data, Xiang et al. (2018) reconstructed the backbone phylogeny of Ajugoideae. Their analyses confirmed the findings of Cantino et al. (1999), supporting the non-monophyly of the traditional circumscribed *Caryopteris* and monophyly of the re-defined *Schnabelia*. Furthermore, Xiang et al. (2018) divided Schnabelia into two sections. Sect. Schnabelia consists of two species (*S.oligophylla* and *S.tetrodonta*), characterised by perennial herb, 4-winged stems, caducous leaves and nutlets puberulent. In contrast, sect. Cylindricaulis C.L.Xiang & H.Peng includes three species (*S.aureoglandulosa*, *S.nepetifolia* and *S.terniflora*), characterised by subshrubs, nearly terete stems without wings, persistent leaves and densely hirsute nutlets with distinct reticulate venation.

As an endemic genus, all species of *Schnabelia* are distributed across central, northern, southern and south-western China, primarily inhabiting the slopes of mountains within subtropic forests (Wu and Li 1977; Xiang et al. 2018; Wang 2019; Zhao et al. 2021; Wang and Hong 2022). During a field expedition in Sichuan Province in 2019, we discovered an intriguing species of *Schnabelia*. Through comparative morphological studies and molecular phylogenetic analyses using plastid and nuclear DNA sequences, we determined this species as a new species of the genus *Schnabelia* and describe and illustrate it here.

## ﻿Material and methods

### ﻿Plant materials

Two populations of the potential new species were collected from Jiuzhaigou County, north-eastern Sichuan, in July 2019. Field photographs and phenological data were recorded during the collection. Fresh leaves were collected and dried with silica-gel for DNA extraction (Chase and Hills 1991). Mature nutlets were also collected in the field. Voucher specimens are deposited in the Herbarium of Kunming Institute of Botany (KUN) and Herbarium of Chengdu Institute of Biology (CDBI), Chinese Academy of Sciences.

### ﻿Morphological study

Morphological characteristics of stems, leaves, inflorescences and flowers of the new species were photographed and measured from living plants and dried specimens. Nutlets were photographed using a Keyence VHX-700F Digital Microscope (Keyence, Osaka, Japan). All morphological features were described following the terminology of Li and Hedge (1994). In addition, specimens of all *Schnabelia* deposited in BM, CDBI, IBK, IBSC, K, KUN, NAS, NWTC, PE, SM, SZ, WCSBG and WUK were thoroughly examined for morphological comparison.

### ﻿Molecular phylogenetic analyses

In order to clarify the systematic position of the new species within *Schnabelia*, we reconstructed the phylogeny of *Schnabelia* using seven DNA markers, as employed by Xiang et al. (2018): the nuclear ribosomal internal and external transcribed spacers (ITS and ETS) and five plastid DNA regions (*matK*, *rbcL*, *rps16*, *trmL-trnF* and *trnH-psbA*). Total Genomic DNA was extracted from silica-gel dried leaf tissue of two individuals of the new species using the modified CTAB method (Doyle and Doyle 1987). Primers details, Polymerase Chain Reaction (PCR) amplification, sequencing and PCR protocols followed those described by Xiang et al. (2018). In total, 112 accessions, representing all five recognised species and the potential new species of the *Schnabelia* were included as the ingroup. Six species from Teucrieae (viz. *Rubiteucris* Kudô and *Teucrium* L.) were selected as outgroup, based on previous phylogenetic frameworks (Xiang et al. 2018; Zhao et al. 2021). The accession numbers for the newly-sequenced samples have been deposited in the GenBank under PQ581109–PQ581112, PQ588101–PQ588102 and PQ594948–PQ594955 and voucher information for all species is provided in the Table 1.

**Table 1.** Information of the samples used for phylogenetic inference in this study. Sequences newly generated in this study are highlighted in bold font.

Taxa	Voucher	Location	*matK*	*rbcL*	*rps16*	*trnL-trnF*	*trnH-psbA*	ITS	ETS
*Schnabeliatetrodonta* (Y.Z. Sun) C.Y. Wu & C. Chen 1	Xiang et al., 352(KUN)	Chongqing, Nanchuan	MF801745	MF801799	MF801857	MF801949	MF801899	MF801694	MF801659
*Schnabeliatetrodonta* (Y.Z. Sun) C.Y. Wu & C. Chen 2	Yang et al., 001(KUN)	Yunnan, Menglun	MF801746	MF801800	MF801858	MF801950	MF801900	MF801695	MF801660
*Schnabeliaoligophylla* Hand.-Mazz. 1	Xiang et al., 353(KUN)	Chongqing, Nanchuan	MF801738	MF801792	MF801850	MF801942	MF801892	MF801687	MF801652
*Schnabeliaoligophylla* Hand.-Mazz. 2	Xiang et al., sn.(Cultivate)	Hubei, Enshi	MF801739	MF801793	MF801851	MF801943	MF801893	MF801688	MF801653
*Schnabeliaoligophylla* Hand.-Mazz. 3	Liu Yanchun, 003(Cultivate)	Shanghai	MF801740	MF801794	MF801852	MF801944	MF801894	MF801689	MF801654
*Schnabelianepetifolia* (Benth.) P.D. Cantino 1	Xiang et al. 590(Cultivate)	Jiangsu, Nanjing	MF801735	MF801789	MF801847	MF801939	MF801889	MF801684	MF801649
*Schnabelianepetifolia* (Benth.) P.D. Cantino 2	Liu Yanchun, 002(KUN)	Shanghai	MF801736	MF801790	MF801848	MF801940	MF801890	MF801685	MF801650
*Schnabelianepetifolia* (Benth.) P.D. Cantino 3	P. D. Cantino, 1428(KUN)	American	MF801737	MF801791	MF801849	MF801941	MF801891	MF801686	MF801651
*Schnabeliaterniflora* (Maxim.) P.D. Cantino 1	Xiang et al., 079(Cultivate)	Yunnan, Kunming	MF801741	MF801795	MF801853	MF801945	MF801895	MF801690	MF801655
*Schnabeliaterniflora* (Maxim.) P.D. Cantino 2	Fang et al., fw11146(KUN)	Gansu, Kang	MF801742	MF801796	MF801854	MF801946	MF801896	MF801691	MF801656
*Schnabeliaterniflora* (Maxim.) P.D. Cantino 3	Xiang et al., 749(KUN)	Hubei, Shengnongjia	MF801743	MF801797	MF801855	MF801947	MF801897	MF801692	MF801657
*Schnabeliaterniflora* (Maxim.) P.D. Cantino 4	Liu Yanchun, 001(KUN)	Shanghai	MF801744	MF801798	MF801856	MF801948	MF801898	MF801693	MF801658
*Schnabeliaaureoglandulosa* (Vaniot) P.D. Cantino 1	Xiang et al., 345(KUN)	Chongqing, Nanchuan	MF801733	MF801787	MF801845	MF801937	MF801887	MF801682	MF801647
*Schnabeliaaureoglandulosa* (Vaniot) P.D. Cantino 2	Liu et al., 3986(KUN)	Yunnan, Funing	MF801734	MF801788	MF801846	MF801938	MF801888	MF801683	MF801648
***Schnabeliajiuzhaigouensis* C. Liu, F. Zhao & C.L. Xiang sp. nov. 1**	**Zhang et al., 19CS18246 (KUN)**	**Sichuan, Jiuzhaigou**	**PQ594948**	**PQ594950**	**PQ581109**	**PQ581111**	**PQ594952**	**PQ588101**	**PQ594954**
***Schnabeliajiuzhaigouensis* C. Liu, F. Zhao & C.L. Xiang sp. nov. 2**	**Zhang et al., 19CS18370 (KUN)**	**Sichuan, Jiuzhaigou**	**PQ594949**	**PQ594951**	**PQ581110**	**PQ581112**	**PQ594953**	**PQ588102**	**PQ594955**
**Outgroup**									
*Teucriumornatum* Hemsl.	Xiang et al., 332(KUN)	Chongqing, Wuxi	MF801748	MF801803	MF801862	MF801952	MF801902	MF801696	MF801662
*Teucriumquadrifarium* Buch.-Ham. ex D. Don	Xiang et al., s.n.(KUN)	Yunnan, Funing	MF801749	MF801804	MF801863	MF801953	MF801903	MF801697	MF801663
*Teucriumviscidum* Blume	Liu et al., 3083(KUN)	Gansu, Wenxian	HQ839703	MF801805	MF801864	MF801954	FJ513102	MF801698	MF801664
*Teucriumbidentatum* Hemsl.	Xiang et al., 336(KUN)	Chongqing, Jinfoshan	MF801747	MF801802	MF801861	MF801951	MF801901	AF477790	MF801661
*Rubiteucrispalmata* (Benth. ex Hook. f.) Kudô	Liu et al., 2998(KUN)	Sichuan, Yanyuan	MF801730	MF801784	MF801842	MF801934	MF801884	MF801679	MF801644
*Rubiteucrissiccanea* (W.W. Sm.) P.D. Cantino 2	Xiang et al., 847(KUN)	Yunnan, Yuanjiang	MF801731	MF801785	MF801843	MF801935	MF801885	MF801680	MF801645
*Rubiteucrissiccanea* (W.W. Sm.) P.D. Cantino 1	Xiang et al., 365(KUN)	Yunnan, Songming	MF801732	MF801786	MF801844	MF801936	MF801886	MF801681	MF801646

Sequences were aligned using MAFFT V. 7.505 (Katoh and Standley 2013), with any ambiguous sites adjusted manually in PhyDE v.0.9971(Müller et al. 2010). Two datasets were constructed for phylogenetic reconstruction. The first dataset (5CP) combined *matK*, *rbcL*, *rps16*, *trnL-trnF* and *trnH-psbA*, while the second dataset (2NR) combined nrITS and ETS sequences.

Phylogenetic analyses were conducted using Maximum Likelihood (ML) and Bayesian Inference (BI) methods. The ML analysis was run on the CIPRES Science Gateway web server (http://www.phylo.org/; Miller et al. 2010) with RAxML V. 8.2.10 (Stamatakis 2014) under the GTR + *Γ* substitution model. The partitioned model (-q) was used for the concatenated plastid data, bootstrap iterations (-# | -N) set to 1000 and other parameters followed default settings. The BI analysis was performed in MrBayes 3.2.6 (Ronquist et al. 2012). The best-fit substitution model of each region was selected independently under the Akaike Information Criterion (AIC) using jModelTest2 (Darriba et al. 2012). For each Bayesian analysis, four MCMC chains (three heated, one cold) were run simultaneously for 20 million generations, starting with a random tree and sampled every 1000 generations. Chain convergence and estimated sample size (ESS) parameters were assessed with Tracer v.1.7.0 (Rambaut et al. 2018), with the first 25% of the trees obtained in BI analysis being discarded as burn-in and then posterior probabilities (PP) were determined from the posterior distribution. The phylogenetic trees were visualised in FigTree v.1.4.3 (Rambaut 2014; http://tree.bio.ed.ac.uk/software/figtree/).

## ﻿Results and discussion

A total of 14 DNA sequences were newly generated in this study, including the seven DNA regions from two accessions of the potential new species. The combined plastid dataset had an aligned length of 4,689 bp (1,136 bp for *matK*, 1,258 bp for *rbcL*, 839 bp for *trnL*-*trnF*, 1008 bp for *rps16* and 448 bp for *trnH*-*psbA*), while the combined nuclear dataset was 992 bp (532 bp for ITS, 460 bp for ETS), respectively.

**Figure 1.**
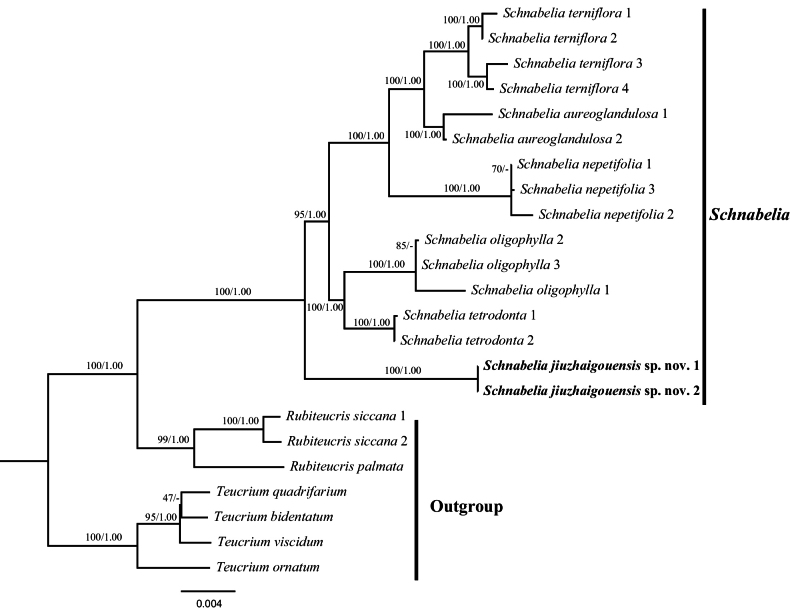
Phylogenetic relationship of *Schnabelia*, based on the (5CPDNA) dataset. The support values (BS/PP) indicated at branches. BS values < 50% and PP support < 90% indicated by -. The outgroup and recognised groups are marked in the right bar.

**Figure 2.**
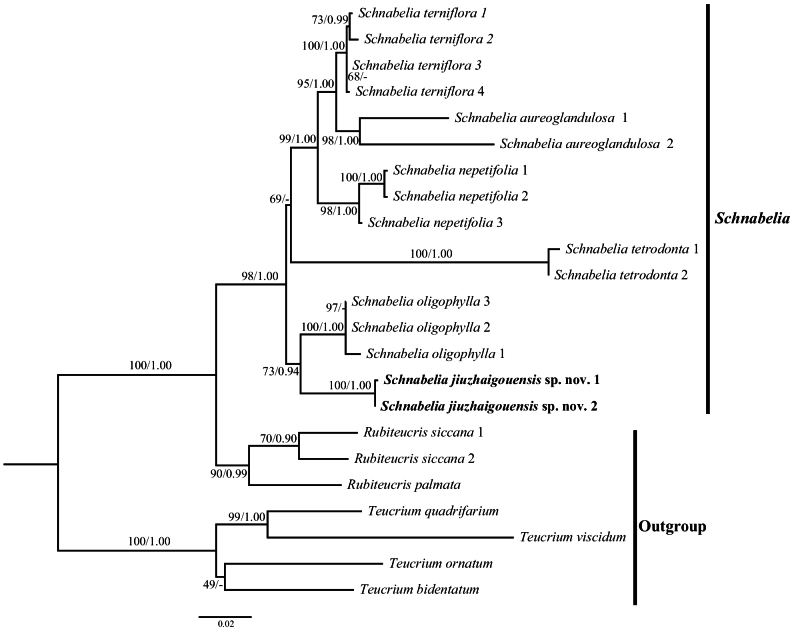
Phylogenetic relationship of *Schnabelia*, based on the (2NR) dataset. The support values (BS/PP) indicated at branches. BS values < 50% and PP support < 90% indicated by -. The outgroup and recognised groups are marked in the right bar.

The phylogenetic topologies from Maximum Likelihood (ML) and Bayesian Inference (BI) analyses were congruent, so only the ML trees were provided for discussion (Figs 1, 2). Both analyses strongly supported the monophyly of *Schnabelia* (ML-BS = 100%, BI-PP = 1.00; all values follow this order hereafter), with the genus *Rubiteucris* found to be sister to *Schnabelia* (100%/1.00). The phylogenetic tree constructed in this study is largely consistent with previous studies, based on the plastid DNA (Li et al. 2016; Xiang et al. 2018; Zhao et al. 2021).

Within *Schnabelia*, the two accessions of the new species formed a clade (Fig. 1: 100/1.00; Fig. 2: 100/1.00). In the plastid phylogenetic tree, the two individuals of the new species formed a subclade sister to the remaining five species and relationships amongst these species were consistent with previous studies (Xiang et al. 2018). In addition, each species formed a monophyletic subclade (Fig. 1).

However, the nuclear phylogenetic tree revealed three subclades. The first subclade included two accessions of the new species and three individuals of *Schnabeliaoligophylla*, forming a moderately supported subclade (Fig. 2. 73/0.94). The second subclade consisted of two individuals of *S.tetrodonta* (Fig. 2. BS/PP = 69/-), while the third subclade (BS/PP = 99/1.00) consisted of the remaining three species (*S.nepetifolia*, *S.aureoglandulosa* and *S.terniflora*). The relationship amongst species within this subclade were consistent with those found in the plastid phylogenetic tree.

Morphologically, based on its non-winged stem and persistent leaves, the potential new species should be placed within sect. Cylindricaulis. It is most similar to *Schnabeliaterniflora*, but can be distinguished by differences in habit, leaf characteristics, corolla shape and the surface ornamentation of the nutlets. Detailed morphological differences between the two species are summarised in Table 2.

**Table 2.** Morphological comparisons between *Schnabeliajiuzhaigouensis* and *S.terniflora*.

Characters	*S.jiuzhaigouensis*	*S.terniflora*
Habit	perennial herb	shrub
Lamina	ovate to lanceolate-ovate leaf blades, 2–8 × 1.5–4 cm, lateral veins 5–8	lanceolate-oblong to ovate, 1.5–4 × 1–3 cm), lateral veins 3–6
Corolla	upper lip and lateral lobes of lower lip oblong, middle lobe cuneate, corolla outside puberulent with non-glandular	upper lip and lateral lobes of lower lip broadly obovate, middle lobe subrounded, corolla outside puberulent with glandular
Nutlets	puberulent, without reticulate veins	densely hirsute, with distinctly reticulate veins

### ﻿Taxonomic treatment

#### ﻿ Schnabelia jiuzhaigouensis

Taxon classificationPlantaeLamialesLamiaceae

C.Liu, F.Zhao & C.L.Xiang sp. nov.

81D3F315-638B-5FF2-A0D1-718F4EA3648C

urn:lsid:ipni.org:names:77357312-1

Fig. 3

##### Type.

China • Sichuan Province, Jiuzhaigou County, in the vicinity of Wujiao Ranger Station of Wujiao Nature Reserve, the wet area along the stream, under the mixed forest, 32°54′42″N, 104°14′38″E, 2599 m a.s.l., 12 July 2019, flowering, *Ting Zhang, C. Liu, H. Jiang, Y.L. He & C.H. Li 19CS18246* (holotype: KUN 1630399!), (isotype: KUN 1630400!; CDBI0290980!)

**Figure 3.**
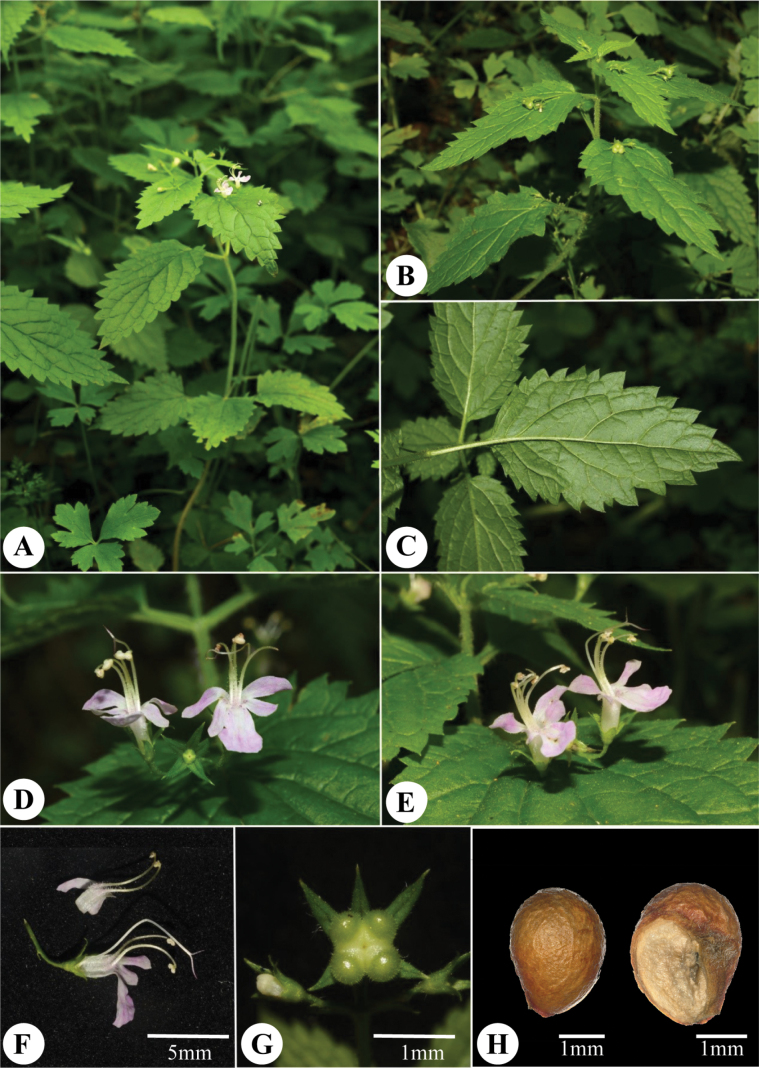
Morphology of *Schnabeliajiuzhaigouensis*. **A, B** habitat **C** leaf morphology **D** frontal view of inflorescence **E** lateral view of inflorescence **F** lateral view of flowers and filaments (Scale bar: 5 mm) **G** frontal view of calyces (Scale bar: 1 mm) **H** surface of the nutlets. (Scale bars: 1 mm) (Photo by Cheng Liu).

##### Diagnosis.

*Schnabeliajiuzhaigouensis* is most similar to *S.terniflora*, but can be distinguished by several morphological differences. *Schnabeliajiuzhaigouensis* is a perennial herb (vs. shrub), with ovate to lanceolate-ovate leaf blades, 2–8 × 1.5–4 cm (vs. lanceolate-oblong to ovate, 1.5–4 × 1–3 cm in *S.terniflora*), the lateral veins on each side of the mid-rib number 5–8 (vs. 3–6 in *S.terniflora*).The upper lip and lateral lobes of the lower lip are oblong in *S.jiuzhaigouensis* (vs. broadly obovate in *S.terniflora*) and the middle lobe is cuneate (vs. subrounded). The corolla is puberulent on the outside, but non-glandular (vs. puberulent and glandular in *S.terniflora*) and the nutlets puberulent (vs. densely hirsute), with *S.jiuzhaigouensis* lacking reticulate veins on the nutlets (vs. with distinctly reticulate veins in *S.terniflora*). The differences between the new species and *S.terniflora* are summarised in Table 2.

##### Description.

Perennial herbs, erect, 20–60 cm tall. Stems 4-angled and puberulent, occasionally branched from base; internodes 1–11 cm long, puberulent. Leaves opposite, petiole 0.3–2.5 cm long, puberulent; leaf blades ovate to lanceolate-ovate, papery, 2–8 × 1.5–4 cm, puberulent on both surfaces, hairs denser along veins abaxially, base broadly cuneate to rounded or subcordate, apex acuminate, margin serrate, lateral veins 5–8 on each side of the mid-rib. Inflorescences axillary cymes, 1–3 flowered; peduncle slender, (0.5)1.5–3.5 cm, puberulent; pedicels 0.2–1 cm long; bracts lanceolate, ca. 1–2 mm long, glabrous or sparsely hairy, bracteoles lanceolate, 0.5–1 mm long. Calyx campanulate, 10-veined, outside sparsely pubescent; tube 1–1.5 mm long, lobes 5, lanceolate, ca. 2–3 × 1 mm, margin entire, apex acuminate. Corolla pink, 2-lipped, outside sparsely puberulent; tube ca. 5 mm long, both surfaces puberulent; upper lip 2-lobed, lobes oblong, ca. 3–3.5 × 1 mm; lower lip 3-lobed, lateral lobes oblong, ca. 2.5–3 × 1 mm, middle lobe larger, cuneate, 5–6 × 3–4 mm. Stamens and style strongly exserted; stamens 4, inserted near corolla throat, filaments 6–8 mm, basally hairy; style 13–16 mm, glabrous, apex subequally 2-lobed. Ovary pubescent. Nutlets 4, ovoid, puberulent, ca. 3 × 2 mm, without reticulate veins.

##### Phenology.

Flowering from June to July, fruiting from July to September.

##### Distribution and habitat.

Currently, *S.jiuzhaigouensis* is known from two locations in Wujiao Xiang, Jiuzhaigou County, Sichuan, China. It grows under the forest together with species of *Piceabrachytyla* (Franch.) E.Pritz. (Pinaceae), *Rosaomeiensis* Rolfe (Rosaceae), *Impatiensundulata* Y.L. Chen & Y.Q. Lu (Balsaminaceae) and *Mimulus* L., at elevations from 2500 m to 2600 m.

##### Etymology.

The specific epithet ‘*jiuzhaigouensis*’ is derived from the type locality Jiuzhaigou County.

##### Chinese name.

Jiǔ Zhài Gōu Sì Léng Căo (Chinese pronunciation); 九寨沟四棱草 (Chinese name).

##### Conservation status.

So far, two small populations of *Schnabeliajiuzhaigouensis* have been observed in the Wujiao Nature Reserve. These populations are geographically close enough to be considered part of a single location. Further detailed investigation of the same habitats is necessary to gain a better understanding of the species’ distribution, abundance and potential threats. Therefore, this species is currently classified as Data Deficient (DD) according to the IUCN Red List Categories and Criteria (IUCN 2024).

##### Additional specimens examined.

*Schnabeliajiuzhaigouensis* (paratypes). China • Sichuan: Jiuzhaigou County, Jiawuchi scenic spot of Wujiao Xiang, under the forest with the species of *Piceabrachytyla*, 32°58′29″N, 104°09′29″E, 2551 m a.s.l., 15 July 2019, flowering, *Ting Zhang, C. Liu, H. Jiang, Y.L. He & C.H. Li 19CS18370* (KUN!).

##### Specimens of *S.terniflora* examined.

**China, Gansu** • Kang County, Qujiagou, 09 Aug 2011, *W. Fang et al. fw11146* (KUN), Jia’an Town, Yuanjiawan, 01 May 1963, *Y.Q. He & C.L. Tang 145* (WUK) • Tanchang County, Hanban County, 11 Jul 1951, *T.P. Wang 14296* (PE) • Wen County, Motianling, Baishui Jiang Nature Reserve, 07 May 2007, *D.E. Boufford et al. 37468* (PE), Bikou Town, Yinchanggou, 05 Jul 1964, *Q.X. Li & X.C. Zhao 2037* (NWTC), Fanba Town, Heiyinggou, 19 Aug 1976, *J.X. Yang 3730* (IBSC); Zhouqu County, Gongbagou, 16 Jul 1998, *Baishuijiang Exped*. 064 (PE); **Hubei** • Shennongjia, on the way from Xinhua to Xiaoluoxi, 11 Jun 2013, *C.L. Xiang et al. 749* (KUN); **Shaanxi** • Mian County, Fangjiaba, 12 Apr 1942, *K.T. Fu 3529* (PE), Liushuying, 15 Apr 1938, *T.P. Wang 9065* (WUK); Nanzheng County, Near Xiaoba Village, 21 Apr 1973, *X.X. Hou 496* (WUK); Near Xiaotai Mountain, 07 May 1956, *Huanghe Exped. 521* (WUK); **Sichuan** • Dujiangyan City, Shichangwan, 19 Apr 1952, *Z. He 12165* (PE), Erwang Temple, 27 Apr 1987, *D.Z. Fu et al. 87*-*0276* (WCSBG), Qingcheng Mountain, 14 Apr 1930, *F.T. Wang 20395* (WUK), Yulei Mountain. 23 Aug 1987, *Z.Y. Li et al. 87*-*1962* (WCSBG); Maerkang County, 11 May 1957, *X. Li 70613* (IBSC), Shuadan Road, 17 May 1957, *X. Li 70662* (NAS) • Mao County, Fushun Town, Gangou, 1 Aug 1975, *Sichuan Veget. Exped. 8680* (CDBI) • Nanping (Jiuzhaigou) County; 26 Oct 1937, *T.P. Wang 7974* (PE), 17 May 1979, *Anonymous 0098* (SM) • LangZhai Village, 15 Jul 1983, *Sichuan Veget. Exped. 30420* (CDBI).

### ﻿The diagnostic key for *Schnabelia*

**Table:** 

1	Stems 4-winged; leaves caducous, blade pubescent or subglabrous, but lacking subsessile glandular trichomes; calyx deeply lobed to base, teeth 2× as long as tube	**sect. Schnabelia (2)**
–	Stems nearly terete, not winged; leaves persistent, blade with subsessile glandular trichomes as well as non-glandular hairs; calyx lobed nearly 1⁄2 its length, teeth as long as tube	**sect.Cylindricaulis (3)**
2	Cymes usually reduced to 1 flower; peduncle longer than 7 mm; calyx 5-dentate	***Schnabeliaoligophylla***
–	Cymes usually 1–3-flowered; peduncle less than 2 mm; calyx 4-dentate	***S.tetrodonta***
3	Leaf blade irregularly sharply serrate with 1–3 teeth per side, those subtending cymes subentire; ovary and nutlets with yellow hairs	***S.aureoglandulosa***
–	Leaf blade, including those subtending cymes, regularly serrate to crenate with 4–10 teeth or lobes per side; ovary and nutlets with white hairs	**4**
4	Leaf blade crenate with 4–6 rounded lobes per side; flowers always solitary	***S.nepetifolia***
–	Leaf blade serrate with more than 6 sharp teeth per side; flowers mostly in (2- or) 3–5-flowered cymes, rarely solitary	**5**
5	Perennial herbs, lateral veins 5–8, upper lip and lateral lobes of lower lip oblong, middle lobe cuneate, nutlets puberulent and without reticulate veins	***S.jiuzhaigouensis***
–	Shrub, lateral veins 3–6, upper lip and lateral lobes of lower lip broadly obovate, middle lobe subrounded, nutlets densely hirsute and with distinctly reticulate veins	***S.terniflora***

## Supplementary Material

XML Treatment for Schnabelia jiuzhaigouensis

## ﻿Additional information

### Author ORCIDs

Fei Zhao https://orcid.org/0000-0001-7328-2262

Peng-Wei Gao https://orcid.org/0009-0009-0475-9739

Ting Zhang https://orcid.org/0000-0003-0939-8468

Cheng Liu https://orcid.org/0000-0001-7146-2542

Chun-Lei Xiang https://orcid.org/0000-0001-8775-6967

### Data availability

All of the data that support the findings of this study are available in the main text.
